# Adherence to evidence-based implementation of antimicrobial treatment guidelines among prescribers in sub-Saharan Africa: a systematic review and meta-analysis

**DOI:** 10.1186/s40545-023-00634-0

**Published:** 2023-11-07

**Authors:** Minyahil Tadesse Boltena, Mirkuzie Woldie, Yibeltal Siraneh, Veronica Steck, Ziad El-Khatib, Sudhakar Morankar

**Affiliations:** 1 Ethiopian Evidence Based Health Care Centre: A Joanna Briggs Institute Center of Excellence, Faculty of Public Health, Institute of Health, Jimma University, Jimma, Ethiopia; 2 Knowledge Translation Division, Knowledge Management Directorate, Armauer Hansen Research Institute, Ministry of Health, Addis Ababa, Ethiopia; 3 University of Technology Sydney, Sydney, Australia; 4 Department of Pharmacology and Therapeutics, Faculty of Life Sciences, McGill University, McGill, Montreal Canada; 5 Global Public Health Department, Karolinska Institute, Stockholm, Sweden; 6 World Health Programme, Université du Québec en Abitibi-Témiscamingue (UQAT), Rouyn-Noranda, Quebec, Canada

**Keywords:** Adherence, Antibiotics prescription, Evidence-based medicine and infection, Standard treatment guidelines, Sub-Saharan Africa

## Abstract

**Background:**

Adherence to evidence-based standard treatment guidelines (STGs) enable healthcare providers to deliver consistently appropriate diagnosis and treatment. Irrational use of antimicrobials significantly contributes to antimicrobial resistance in sub-Saharan Africa (SSA).  The best available evidence is needed to guide healthcare providers on adherence to evidence-based implementation of STGs. This systematic review and meta-analysis aimed to determine the pooled prevalence of adherence to evidence-based implementation of antimicrobial treatment guidelines among prescribers in SSA.

**Methods:**

The review followed the JBI methodology for systematic reviews of prevalence data. CINAHL, Embase, PubMed, Scopus, and Web of Science databases were searched with no language and publication year limitations. STATA version 17 were used for meta-analysis**.** The publication bias and heterogeneity were assessed using Egger’s test and the *I*^2^ statistics. Heterogeneity and publication bias were validated using Duval and Tweedie's nonparametric trim and fill analysis using the random-effect analysis. The summary prevalence and the corresponding 95% confidence interval (CI) of healthcare professionals’ compliance with evidence-based implementation of STG were estimated using random effect model. The review protocol has been registered with PROSPERO code CRD42023389011. The PRISMA flow diagram and checklist were used to report studies included, excluded and their corresponding section in the manuscript.

**Results:**

Twenty-two studies with a total of 17,017 study participants from 14 countries in sub-Saharan Africa were included. The pooled prevalence of adherence to evidence-based implementation of antimicrobial treatment guidelines in SSA were 45%. The pooled prevalence of the most common clinical indications were respiratory tract (35%) and gastrointestinal infections (18%). Overall prescriptions per wards were inpatients (14,413) and outpatients (12,845). Only 391 prescribers accessed standard treatment guidelines during prescription of antimicrobials.

**Conclusions:**

Healthcare professionals’ adherence to evidence-based implementation of STG for antimicrobial treatment were low in SSA. Healthcare systems in SSA must make concerted efforts to enhance prescribers access to STGs through optimization of mobile clinical decision support applications. Innovative, informative, and interactive strategies must be in place by the healthcare systems in SSA to empower healthcare providers to make evidence-based clinical decisions informed by the best available evidence and patient preferences, to ultimately improving patient outcomes and promoting appropriate antimicrobial use.

**Supplementary Information:**

The online version contains supplementary material available at 10.1186/s40545-023-00634-0.

## Background

The World Health Organization (WHO) declared antimicrobial resistance (AMR) as a growing global health security and development threat that undermines the effectiveness of antimicrobial agents, threatening the ability to treat common microbial infections [1]. AMR poses a significant economic risk as it leads to higher patient care costs due to prolonged hospitalization, wastage of clinical and human resources, and a demand for the development of novel antimicrobial therapeutics [2, 3].

If preventative measures are not taken, the threat of will persist and result in a depletion of resources and an increase in morbidity and mortality on a global scale [4, 5]. Low- and middle-income countries (LMICs) suffer greater consequences due to insufficient funding, preventing access to costly second or third-line treatment alternatives [6]. AMR is often a result of misuse and overuse by the patient which is often attributable to inappropriate prescription by the health care provider (HCP) [7].

To combat inappropriate antimicrobial use, the development of standard treatment guidelines (STGs) has been included as part of the WHO’s Global Action Plan initiative; with this implementation, the WHO aims to set guidelines for the purchasing and prescription of antimicrobial medicine [8, 9]. STGs help to standardize treatment care by guiding the decisions of prescribers and determine the criteria for diagnosis, prevention, management, and treatment of disease [10, 11]. In order for STGs to be effective, they must be continually updated and made accessible to HCPs and patients [12, 13].

Studies have shown that when STGs are adhered to, mortality, morbidity, and the costs of health services related to corresponding illness are reduced [14, 15]. While the potential of STG use is promising, low rates of STG adherence have been documented in LMICs, where less than half of all patients were treated in accordance with STGs [16–18]. Countries in sub-Saharan Africa (SSA) have made use of STGs, either developing their own based on local context, or adopting the WHO guidelines, altering them to be suitable for resource-limited settings [19–23].

Reasons for lack of adherence to STGs include a lack of skilled human resources, costs of the drugs, quality of the STGs, lack of accessibility to the drugs, lack of access to STGs, and inadequate training of prescribers [24, 25]. While the information presented gives us a glimpse of insight into the landscape of adherence to STGs in SSA, this information is not adequate to draw generalizable conclusion regarding patterns of adherence in the area, as most of the current reviews of evidence regarding STG adherence is from HICs [26, 27].

A scoping review that analyzed the overuse of medications in low resource settings found that only 10 out of 139 studies reported drivers of non-adherence-specific antimicrobial treatment guidelines [28, 29]. Thus, best available evidence on antimicrobial prescriptions in the context of SSA is imperative to understand the adherence of healthcare professionals to their respective STGs and the factors which influence compliance to standard antimicrobial treatment guidelines. This knowledge can be used to inform future interventions to improve prescribing behaviors in SSA in line with the WHOs Global Action Plan initiative’s goal to fill important knowledge gap on antimicrobial stewardship [30].

Therefore, this systematic review and meta-analysis aimed to determine the pooled prevalence of adherence to evidence-based implementation of antimicrobial treatment guidelines among prescribers in sub-Saharan Africa. The pooled data output obtained from this review would serve as region-specific and up-to-date evidence that contributes to comprehensive insights into gaps in the implementation of STGs at point of care and provides actionable recommendations for improvement. It would complement and enhance the knowledge gained from previous reviews by offering a more detailed and context-specific analysis.

## Methods

The proposed review were conducted in accordance with the JBI methodology for systematic reviews of prevalence data [31]. The protocol has been registered with PROSPERO (CRD42023389011).

### Search strategy

The database search targeted both published and unpublished studies. There was no language and publication year restrictions. A three-step search strategy were used in this review. First, an initial search of PubMed and CINAHL was undertaken, followed by an analysis of the titles, abstracts, and index terms of the articles. Second, all published and unpublished literature were searched using the identified keywords. Additional file 1: Appendix I shows the full search strategy for all databases. Third, the reference lists of all included primary studies were hand-searched for additional relevant studies. The Embase, Scopus, and Web of Science databases were searched. Moreover, Google Scholar, the Africa CDC and WHO platforms, dissertations, and thesis were searched for gray literature. Study authors were contacted if the full text is unavailable.

### Study selection

Following the search, all identified citations were collated and uploaded into EndNote 20 and duplicates were removed. Descriptive observational and cross-sectional studies were included. Literature was eligible for inclusion if they reported adherence to STGs among prescribers in SSA. Studies which reported the prevalence of healthcare providers adherence to STGs as the main outcome were included. Literature that reported the clinical indications for which antimicrobials were prescribed for, access, availability, frequency of STG use was included. This review included studies conducted in both public and private health facilities in SSA. Protocols, systematic reviews, meta-analysis, randomized controlled trials, and studies conducted in high-income countries were excluded.

**Fig. 1:**
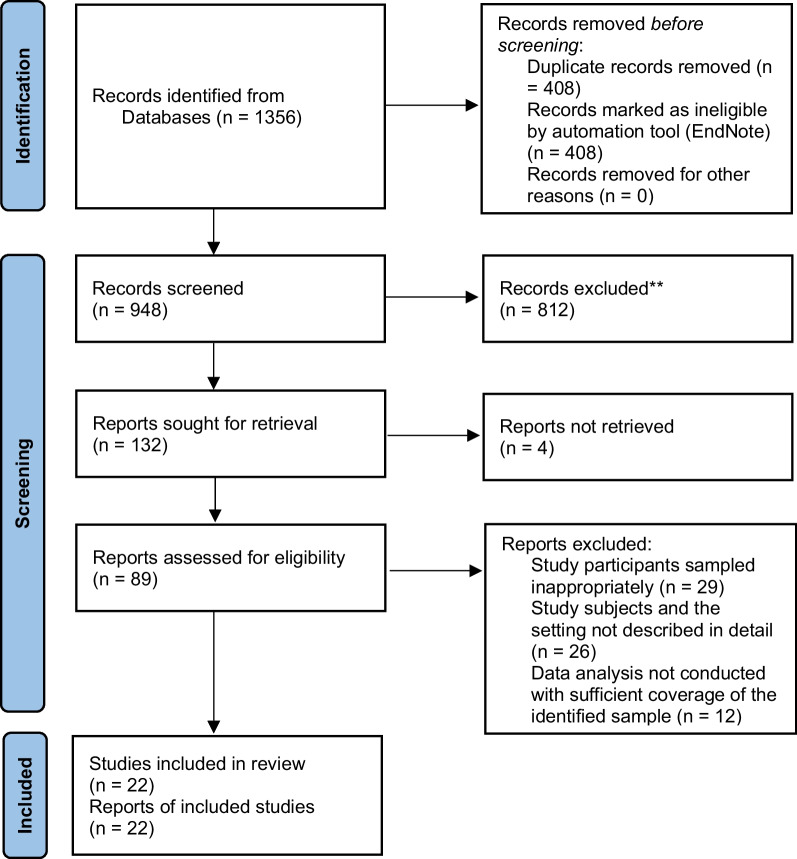
PRISMA flow diagram of included studies: Page et al. [96]

Titles and abstracts were assessed by two independent reviewers (MTB and VS) against the inclusion criteria. The full texts of potentially relevant studies were retrieved and the citation details were imported into the JBI System for the Unified Management, Assessment, and Review of Information (JBI SUMARI) [32]. The full texts of selected citations were assessed in detail against the inclusion criteria by independent reviewers (MTB and VS). Any disagreements that arose between the reviewers at each stage of the selection process were resolved through discussion with a third senior reviewer (SM). The results of the search, study inclusion and exclusion process were reported in full in the final systematic review and presented as a Preferred Reporting Items for Systematic Reviews and Meta-Analyses flow diagram (PRISMA) (Fig. 1) [33]. PRISMA 2020 checklist were used to report each section of the manuscript with its corresponding pages (Additional file 1: Appendix II). Studies that reported healthcare providers adherence to evidence-based antimicrobial treatment guidelines in SSA were included. Literature including healthcare professionals from high-income countries, Middle East and North Africa were excluded. Systematic reviews, clinical trials, meta-analysis were excluded.

### Operational definition

#### Evidence-based implementation of antimicrobial treatment guidelines

Refers to the systematic and rigorous applications of established clinical recommendations for the use of antimicrobial agents in the treatment of infectious diseases [34]. This approach relies on the uptake of the best available scientific evidence, clinical expertise, and patient preferences to inform healthcare providers about the most effective and safest strategies for prescribing antibiotics [35].

#### Adherence to evidence-based implementation of antimicrobial treatment guidelines

Refers to compliance with standard treatment guidelines (STG) for antimicrobial treatment at point of care provided that a consistently correct diagnoses and treatments that limit the irrational use of medicines and the negative health consequences that can occur as a result were in place [36, 37]. Adherence to guidelines denotes the degree of conformity between the knowledge, cognition and/or action of healthcare professionals who are involved in antimicrobial prescription pursuant with the recommendations of a guideline [38, 39]. By adhering to evidence-based guidelines, healthcare providers can optimize patient outcomes, enhance antimicrobial stewardship efforts, and contribute to the overall public health goal of combating antimicrobial resistance [40, 41].

### Data extraction

The data extraction tool was prepared by MTB using excel spreadsheet. The data were extracted from included studies using the data extraction tool prepared by MTB. The tool includes variables such as the name of the author, publication year, study design, data collection period, sample size, study area, and the prevalence of adherence to standard treatment guidelines (STG) among health care providers. In addition, the tool consists of data on the clinical indications, access and availability of STG, frequency of use of STG. MTB and VS extracted the data. YS and SM cross-checked the extracted data for its validity and cleanness. Any disagreements between the reviewers were resolved through discussion with a third reviewer. Authors of the papers were contacted to request missing or additional data as required.

### Assessment of methodological quality

Two independent reviewers critically appraised eligible studies for methodological quality using the JBI critical appraisal checklist for studies reporting prevalence data [42]. Study authors were contacted to request missing or additional data, if required. Any disagreements were resolved through discussion with a third senior reviewer. The results of the critical appraisal were reported in narrative and tabular format. A lower risk of bias (97%) observed after assessment (Table 1).

**Table 1:** Risk bias assessment of included 22 studies

Author and publication year	Q1	Q2	Q3	Q4	Q5	Q6	Q7	Q8	Q9	Total	%
Boonstra et al., 2005	9	9	7	9	8	9	9	9	9	78	96
Boonstra et al., 2002	9	7	9	7	9	9	8	9	8	75	93
Mashalla et al., 2017	8	9	9	9	9	9	9	9	9	80	99
Borchert et al., 1999	9	9	8	8	9	8	8	9	8	76	94
Eticha and Gemechu, 2021	9	9	9	9	8	9	8	7	8	76	94
Prah et al., 2017	9	9	9	9	9	9	9	9	8	80	99
Sefah et al., 2021	7	9	9	9	9	9	9	9	9	79	98
Owusu et al., 2022	9	9	9	8	9	9	9	9	8	79	98
Bosibori , 2016	9	8	9	9	8	9	9	9	9	79	98
Bello, 2021	9	9	9	8	9	9	9	9	9	80	99
Sibande et al., 2022	7	9	9	8	9	9	8	9	9	78	96
Niaz et al., 2020	9	9	8	9	9	9	9	9	9	80	99
Akpabio et al., 2014	9	9	9	8	9	8	9	9	9	79	98
Govender, Suleman and Perumal-Pillay., 2021	9	9	9	8	9	9	9	8	9	79	98
Gasson, Blockman and Willems , 2018	7	9	9	9	9	9	9	9	8	78	96
Mayiste et al., 2017	9	9	9	9	9	9	9	9	9	81	100
Otim et al ., 2021	9	9	9	9		9	9	9	8	79	98
Musa, Harron and Maatoug., 2019	9	9	9	7	8	9	8	9	9	74	91
Wiedenmayer et al., 2021	9	9	9	9	9	9	9	9	9	81	100
Budimu et al., 2020	8	9	9	9	9	9	9	9	8	77	95
Obakiro et al., 2021	9	9	9	9	9	9	9	9	9	81	100
Miyanda, Siame and Chisulo., 2022	9	9	9	9	9	8	8	9	9	76	94
ROB in % is										97%	

### Data synthesis

**Fig. 2:**
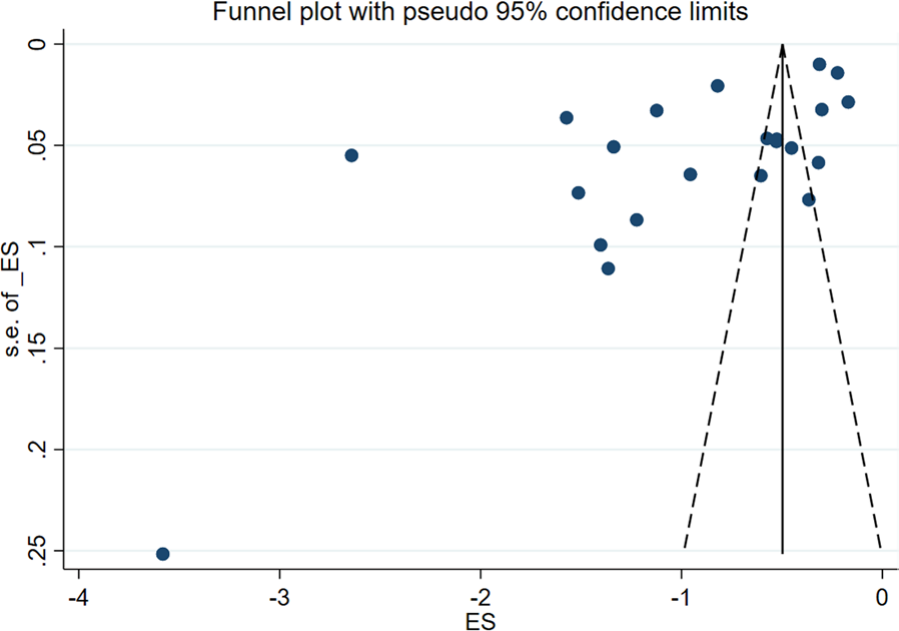
Funnel plot showing symmetric distribution of studies on adherence to evidence-based implementation of antimicrobial treatment guidelines among prescribers in sub-Saharan Africa, 2023

Included studies were pooled in a statistical meta-analysis using STATA version 17.0. Effect size was expressed as a proportion with 95% confidence intervals around the summary estimate. Heterogeneity was assessed using the standard Chi-square *I*^2^ test. A random-effects model using the double arcsine transformation approach were used. Sensitivity analyses were conducted to test decisions made regarding the included studies. Visual examination of funnel plot asymmetry (Fig. 2) and Egger’s regression tests were used to check for publication bias [43]. A Forest plot with 95% CI were computed to estimate the pooled magnitude of adherence to evidence-based antimicrobial treatment guidelines among health care providers in sub-Saharan Africa.

## Results

### Search

Following the automatic removal of 408 literature as duplicates by EndNote 20, a total of 948 articles were obtained from PubMed, CINAHL, EMBASE, Google Scholar, and SCOPUS, and Web of Science databases. At the title/abstract screening phase (*n* = 816) and during the full-article screening (*n* = 110) articles were excluded. Accordingly, 43 studies were eligible for quality assessment. Finally, 22 studies were included in this meta-analysis (Fig. 1).

### Study characteristics

The total sample size of this systematic review was 17,017, ranging from 75 in Nigeria [44] to 3713 in Ghana [45] (Table 1). Three studies were equally reported from Botswana [46–48] Ghana [45, 49, 50], and South Africa [51–53], respectively (Table 2). Two articles were obtained from Namibia [54, 55] and Tanzania [56, 57] (Table 2). Only one literature were obtained from Burkina Faso [58], Ethiopia [59], Kenya [60], Malawi [61], Nigeria [44], South Sudan [62], Sudan [63], Uganda [64], Zambia [65], respectively (Table 2).

**Table 2:** Adherence to standard treatment guidelines (STGs)

Author and publication year	Sample size (n)	Prescriptions adhered to STGs	Country
Boonstra et al., 2005	539	15	Botswana
Boonstra et al., 2002	2994	1318	Botswana
Mashalla et al., 2017	235	60	Botswana
Borchert et al., 1999	313	185	Burkina Faso
Eticha and Gemechu, 2021	217	138	Ethiopia
Prah et al., 2017	338	250	Ghana
Sefah et al., 2021	1929	627	Ghana
Owusu et al., 2022	3713	2714	Ghana
Bosibori , 2016	309	76	Kenya
Bello, 2021	75	52	Nigeria
Sibande et al., 2022	230	194	Malawi
Niaz et al., 2020	1243	994	Namibia
Akpabio et al., 2014	1090	286	Namibia
Govender, Suleman and Perumal-Pillay., 2021	300	177	South Africa
Gasson, Blockman and Willems , 2018	654	144	South Africa
Mayiste et al., 2017	357	201	South Africa
Otim et al ., 2021	316	93	South Sudan
Musa, Harron and Maatoug., 2019	110	80	Sudan
Wiedenmayer et al., 2021	2886	599	Tanzania
Budimu et al., 2020	196	107	Tanzania
Obakiro et al., 2021	4312	307	Uganda
Miyanda, Siame and Chisulo., 2022	385	148	Zambia

Author, publication year	Sample size	Frequency of use						
		Never		Daily	Once a week/often/regularly	Sometimes/occasionally/once in 6 months/once a month	Once in 6 months	Rarely/once a year
Prah et al., 2017	338	7						
Niaz et al., 2020	1243	3		12	7	11	3	1
Govender, Suleman and Perumal-Pillay., 2021	300	0			94	106		5

The most common clinical indications for antibiotics were respiratory tract infection (RTI) reported by eleven studies [46, 49, 50, 52, 55, 56, 59, 61–64], followed by urinary tract infection (UTI) [45, 49, 56, 61], and gastrointestinal disease/infection [52, 62, 64, 65] which were equally indicated by four different studies (Table 3). Three articles described diarrhea [47, 55, 56] as clinical condition (Table 3). Equally two studies reported CNS [61, 65], co-infection [61, 62], Enteric infection [49, 61], Sepsis [61, 65], STIs [52, 53], and Malaria [56, 65] clinical indications for antibiotics, respectively (Table 3).

**Table 3:** Clinical indication for prescription

Author and publication year	Central nervous system	Infection/co-infection	Enteric	Respiratory (upper and lower infection)	Sepsis	Urinary tract infection	Diarrhoea	Gastrointestinal disease/infection	Sexually transmitted infections	Malaria
Boonstra et al., 2005				185			85			
Boonstra et al., 2002										
Mashalla et al., 2017										
Borchert et al., 1999										
Eticha and Gemechu, 2021				217						
Prah et al., 2017			17	56		37				
Sefah et al., 2021				1929						
Owusu et al., 2022						3713				
Bosibori , 2016										
Bello, 2021										
Sibande et al., 2022	73	1	23	89	28	8				
Niaz et al., 2020										
Akpabio et al., 2014				209			118			
Govender, Suleman and Perumal-Pillay., 2021										
Gasson, Blockman and Willems , 2018				182				51	45	
Mayiste et al., 2017									*357*	
Otim et al ., 2021		75		56				63		
Musa, Harron and Maatoug., 2019				110						
Wiedenmayer et al., 2021				72,200%		115	260			519
Budimu et al., 2020										
Obakiro et al., 2021				902				730		
Miyanda, Siame and Chisulo., 2022	26				42			37		18

Public health officers (1616), nurses (731), medical doctors (196), and community health workers (151) were the distribution of STGs prescribers according to profession (Table 4). Educational qualification of prescribers was medical doctor (1676), clinical nurse (679), specialist (617), and internist (100), respectively (Table 4). A total of prescriptions made per ward were 12,845 (outpatient) and 14,413 (inpatient), respectively (Table 4). Three studies [54, 57, 63] reported that only 261 health care providers were aware regarding the use of STGs at point of clinical care (Table 4).

**Table 4:** Access to STGs

Author and publication year	Sample size	Number of participants with access to STGs
Boonstra et al., 2005	539	
Boonstra et al., 2002	2994	
Mashalla et al., 2017	235	
Borchert et al., 1999	313	
Eticha and Gemechu, 2021	217	
Prah et al., 2017	338	5
Sefah et al., 2021	1929	
Owusu et al., 2022	3713	
Bosibori , 2016	309	
Bello, 2021	75	
Sibande et al., 2022	230	
Niaz et al., 2020	1243	35
Akpabio et al., 2014	1090	35
Govender, Suleman and Perumal-Pillay., 2021	300	142
Gasson, Blockman and Willems , 2018	654	
Mayiste et al., 2017	357	
Otim et al ., 2021	316	
Musa, Harron and Maatoug., 2019	110	26
Wiedenmayer et al., 2021	2886	
Budimu et al., 2020	196	148
Obakiro et al., 2021	4312	
Miyanda, Siame and Chisulo., 2022	385	

Only three studies have reported the frequency of STG use by prescribers [49, 51, 54], out of which two articles described that healthcare professionals never used STG [49, 54] (Table 4). Six articles [49, 51, 54, 55, 57, 63] revealed that 391 health care providers accessed STGs during prescription (Table 4). Only two literatures [54, 57] reported that continuous professional development (CPD) training on compliance to STGs were delivered to 213 health care workers (Table 4). The review was conducted on studies that used the cross-sectional designs (Table 4).

### Pooled prevalence of implementation of evidence-based antimicrobial treatment guidelines

**Fig. 3:**
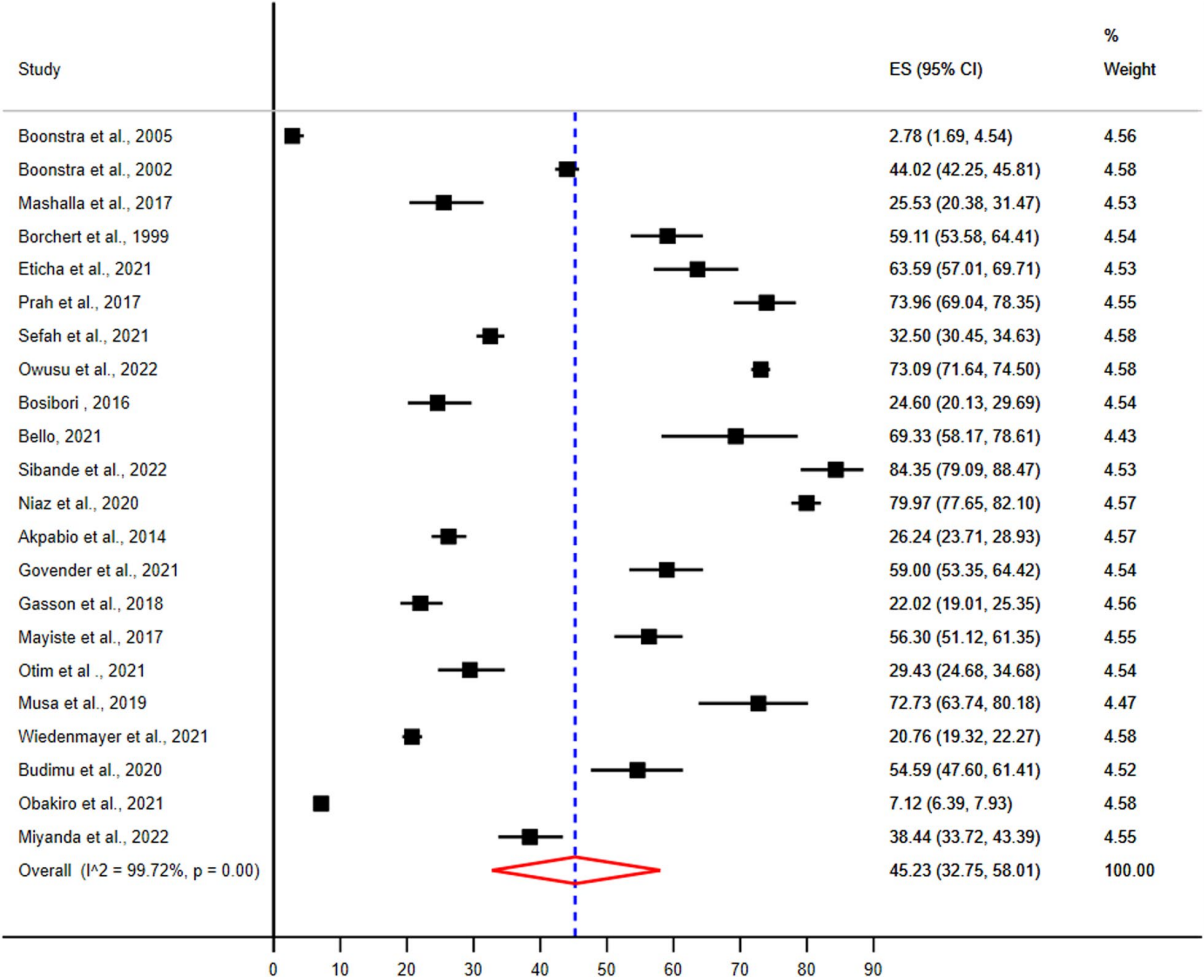
Pooled prevalence of adherence to evidence-based implementation of antimicrobial treatment guidelines among prescribers in sub-Saharan Africa

The pooled prevalence of adherence to evidence-based implementation of antimicrobial treatment guidelines were 45.23% (95% CI 32.75–58.01%) (Fig. 3).

### The pooled prevalence of RTI, UTI, and GI

**Fig. 4:**
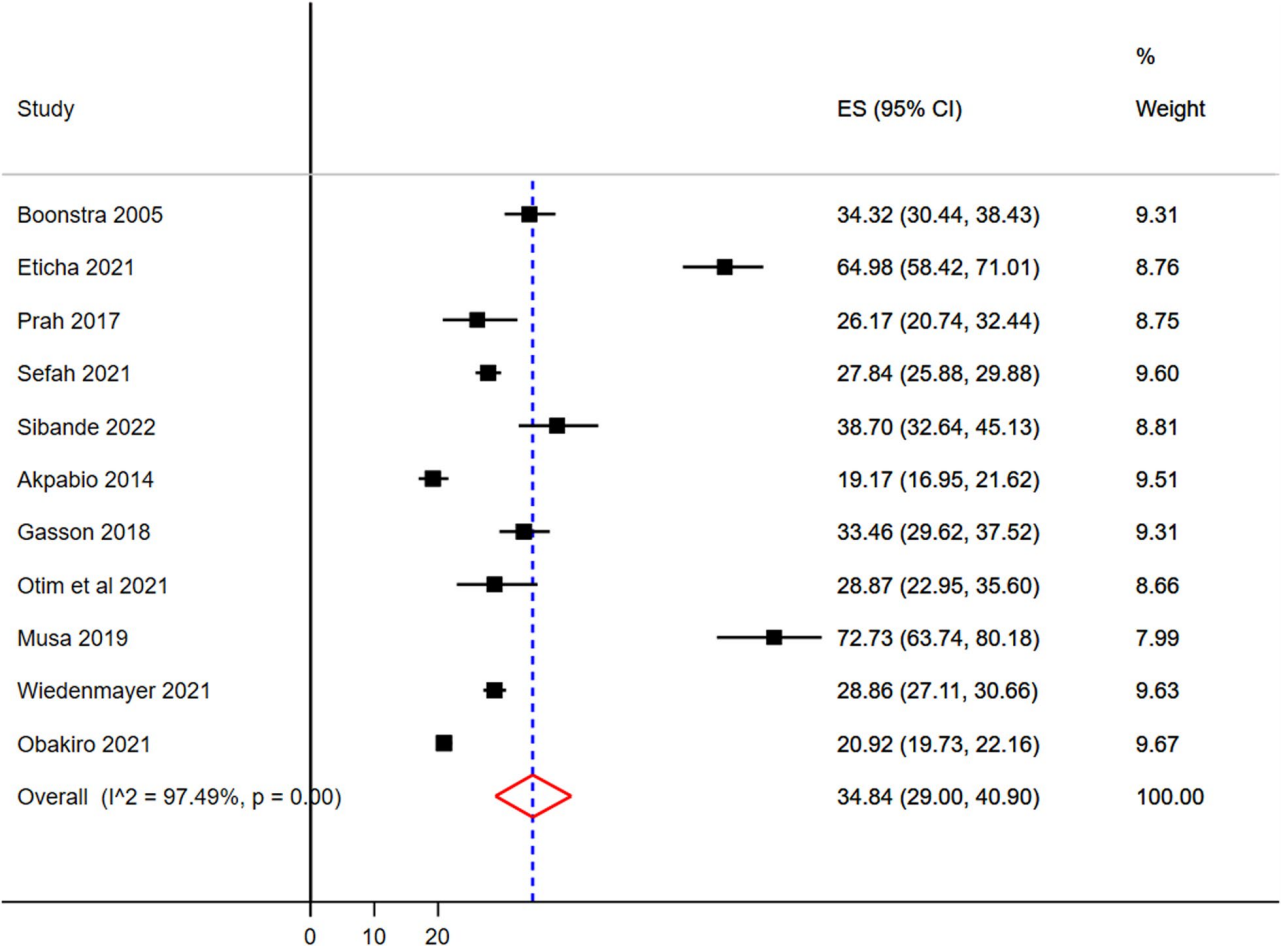
Pooled prevalence of respiratory tract infection as most common clinical indication as prescriptions made in sub-Saharan Africa

**Fig. 5:**
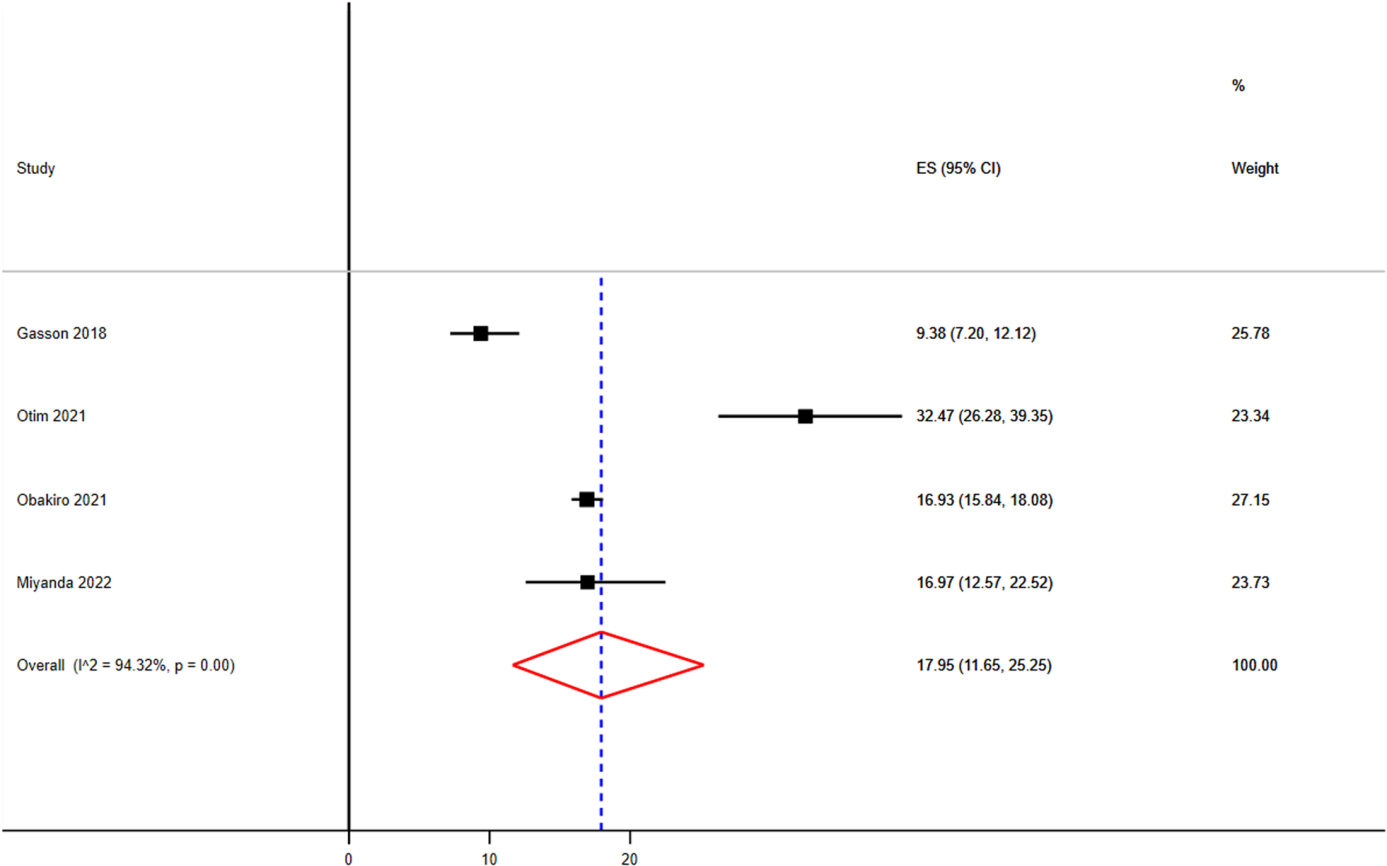
Pooled prevalence of gastrointestinal tract infection as second most common clinical indication as prescriptions made in sub-Saharan Africa

The sample size of RTI ranges from 56 [63] to 902 [64] (Table 3). The pooled prevalence of RTI were 34.84 (95% CI 29.00–40.90%) (Fig. 4). The lowest and the highest infection from gastrointestinal diseases were 37 [65] and 730 [64] (Table 3). The pooled prevalence GI were 17.95% (95% CI 11.65–25.25%) (Fig. 5).

## Discussion

This systematic review and meta-analysis aimed to determine the pooled estimate of implementation of standard treatment guidelines among the prescribers in SSA. A total of 17,017 healthcare professionals who prescribed antimicrobials participated in 22 studies reported from 14 in SSA. The pooled prevalence of adherence to evidence-based antimicrobial treatment guidelines at point of care in SSA were 45%. Lower adherence to evidence-based antimicrobial treatment guidelines can be attributed to healthcare provider-related factors, such as lack of awareness or knowledge about the guidelines [66], doubt regarding their applicability to individual patients [67], limited availability or accessibility of guidelines [68], inadequate resources or infrastructure to support guideline implementation [69], and competing priorities within healthcare settings [70], and patient preferences [71]. Addressing these barriers through targeted educational initiatives, organizational support, and shared decision-making approaches can help improve adherence to evidence-based antimicrobial treatment guidelines and promote optimal patient care [72–74].

This review indicated that only 261 prescribers have awareness regarding the implementation of STG in routine clinical care. Lower awareness among prescribers regarding the use of STG at the point of care can have significant implications for patient care and outcomes [75]. It can lead to variations in clinical practices, with prescribers deviating from evidence-based recommendations [76]. This can result in inconsistent and potentially suboptimal treatment decisions, compromising patient safety and quality of care [77]. Inadequate awareness of guidelines contributes to overuse or inappropriate use of antimicrobial agents, leading to increased healthcare costs, antimicrobial resistance, and adverse drug reactions [78]. Implementation of decision support tools can help improve adherence to guidelines, enhance patient outcomes, and promote the judicious use of antimicrobial treatments [79, 80].

This study revealed that only 391 healthcare providers in SSA accessed STG when they prescribed antimicrobials to patients. Limited access to STG for healthcare providers can lead to variability and inconsistency in prescribing practices [81]. This can result in suboptimal or inappropriate use of antimicrobial agents, potentially compromising patient safety and treatment efficacy [82]. The absence of guidelines can hinder the dissemination of evidence-based recommendations, impeding the implementation of best practices and advancements in antimicrobial stewardship [83, 84]. Healthcare providers may face challenges in keeping up with the rapidly evolving field of infectious diseases and antimicrobial resistance without access to updated guidelines [85].

Healthcare providers in SSA commonly treated cases of respiratory tract infection (35%) and gastrointestinal diseases (18%). Respiratory tract (35%) and gastrointestinal (18%) infections are highly treated clinical indications in SSA. This could be attributed to their significant burden due to easy transmissibility and environmental factors [86, 87].

### Limitations of the study

This systematic review and meta-analysis involved cross-sectional studies that comes with limitations related to causality, selection bias, heterogeneity, and the inability to capture temporal and dynamic trends. To overcome these limitations and obtain a more comprehensive understanding of adherence to implementation of evidence-based STGs, future research could consider incorporating other study designs, such as longitudinal studies or randomized controlled trials, in addition to cross-sectional data.

## Conclusion

Healthcare professionals’ adherence to evidence-based implementation of standard treatment guidelines for antimicrobial treatment were low in sub-Saharan Africa. Healthcare systems in sub-Saharan Africa must make concerted efforts to enhance prescribers access to standard treatment guidelines through the implementation of mobile clinical decision support applications to optimize compliance with standard treatment guidelines. Innovative, informative, and interactive strategies must be in place by the healthcare systems in sub-Saharan Africa to empower healthcare providers to make evidence-based clinical decisions informed by the best available evidence and patient preferences, to ultimately improving patient outcomes and promoting appropriate antimicrobial use.

### Implications for policy and practice

The implementation of evidence-based clinical practice guidelines for antimicrobial treatment involves the systematic integration of the best available evidence into clinical decision-making and patient care [88, 89]. These guidelines are developed based on rigorous research and aim to provide healthcare practitioners with recommendations on the appropriate use of antimicrobial agents for specific infections [90]. The implementation process includes raising awareness about the guidelines, promoting their adoption and acceptance among healthcare professionals, providing education and training on their content and implementation strategies, and addressing barriers and challenges to their implementation [91, 92]. By effectively implementing these guidelines, healthcare systems can optimize antimicrobial therapy, improve patient outcomes, prevent antimicrobial resistance, and ensure the judicious use of these critical medications in sub-Saharan Africa [93–95].

### Supplementary Information

**Additional file 1**: **Appendix I: **Search strategy. **Appendix II**: PRISMA 2020 Checklist. 

## Data Availability

The data sets during and/or analyzed during the current study are available from the corresponding author on reasonable request.

## Abbreviations

AMR: Antimicrobial resistance
AHRI: The Armauer Hansen Research Institute
HCP: Health Care Providers/Professionals
JBI: The Joanna Briggs Institute
JBI SUMARI: The Joanna Briggs Institute’s System for the Unified Management, Assessment, and Review of Information (SUMARI)
LMICs: Low- and middle-income countries
PRISMA: Preferred Reporting Items for Systematic Reviews and Meta-analyses
PROSPERO: International Prospective Registry of Systematic Reviews
SDG: Sustainable development goal
SSA: Sub-Saharan Africa
STGs: Standard Treatment Guidelines
WHO: The World Health Organization
