# Supplementary material for: Adherence to evidence-based implementation of antimicrobial treatment guidelines among prescribers in sub-Saharan Africa: a systematic review and meta-analysis
Source: J Pharm Policy Pract. 2023 Nov 7;16:137. doi: 10.1186/s40545-023-00634-0 (PMC10629154; doi:10.1186/s40545-023-00634-0)
Supplement: Supplementary file 1 — Additional file 1: Appendix I: Search strategy. Appendix II: PRISMA 2020 Checklist. [file 40545_2023_634_MOESM1_ESM.docx]

**Appendix I: Search strategy**

| **PubMed Search strategy** | **Results retrieved** |
| --- | --- |
| ((((((((implementation[MeSH Terms]) OR (adherence, guideline[MeSH Terms])) OR (compliance[MeSH Terms])) AND (implementation[Text Word])) OR (adherence[Text Word])) OR (compliance[Text Word])) AND ((((((drug prescription[MeSH Terms]) OR (drug prescriptions[MeSH Terms])) AND (antibiotic prescription[Text Word])) OR (antimicrobial prescription[Text Word])) OR (drug prescription[Text Word])) OR (drug prescriptions[Text Word]))) AND (((((((((((((adherence, guideline[MeSH Terms]) OR (clinical practice guideline[MeSH Terms])) OR (treatment protocols[MeSH Terms])) OR (compliance[MeSH Terms])))) AND (adherence[Text Word])) OR (compliance[Text Word])) OR (standard treatment guidelines[Text Word])) OR (clinical practice guidelines[Text Word])) OR (implementation[Text Word])) OR (antibiotic use guidelines[Text Word])) OR (antimicrobial use guidelines[Text Word]))) AND (Angola*[tw] OR Benin*[tw] OR Botswana*[tw] or Burkina Faso*[tw] or Burundi*[tw] or Cabo Verde*[tw] or Cameroon*[tw] or Central African Republic*[tw] or Chad*[tw] or Comoros*[tw] or Congo, Democratic Republic of*[tw] or Congo, Republic of*[tw] or Cote d'Ivoire*[tw] or Equatorial Guinea*[tw] or Eritrea*[tw] or Eswatini*[tw] or Ethiopia*[tw] or Gabon*[tw] or Gambia*[tw] or Ghana*[tw] or Guinea*[tw] or Guinea-Bissau*[tw] or Kenya*[tw] or Lesotho*[tw] or Liberia*[tw] or Madagascar*[tw] or Malawi*[tw] or Mali*[tw] or Mauritania*[tw] or Mauritius*[tw] or Mozambique*[tw] or Namibia*[tw] or Niger*[tw] or Nigeria*[tw] or Rwanda*[tw] or Sao Tome and Principe*[tw] or Senegal*[tw] or Seychelles*[tw] or Sierra Leone*[tw] or Somalia*[tw] South Africa*[tw] South Sudan*[tw] or Sudan*[tw] or Tanzania*[tw] or Togo*[tw] or Uganda*[tw] or Zambia*[tw] or Zimbabwe*[tw]) | [**22**](https://pubmed.ncbi.nlm.nih.gov/?term=longquery9c116d7305b0eddfa8c4&sort=) |

| **EMBASE Search strategy** | Result retrieved |
| --- | --- |
| 'prescription'/exp OR 'prescription' OR 'antibiotic prescription'/exp OR 'antibiotic prescription' [AND](https://www-embase-com.proxy.library.emory.edu/) 'implementation'/exp OR 'implementation' OR 'protocol compliance'/exp OR 'protocol compliance' [AND](https://www-embase-com.proxy.library.emory.edu/) 'practice guideline'/exp OR 'practice guideline' OR 'prescribing guideline'/exp OR 'prescribing guideline' OR 'antibiotic use'/exp OR 'antibiotic use' [AND](https://www-embase-com.proxy.library.emory.edu/) 'angola'/exp OR 'benin'/exp OR 'botswana'/exp OR 'burkina faso'/exp OR 'burundi'/exp OR 'cape verde'/exp OR 'cameroon'/exp OR 'central african republic'/exp OR 'chad'/exp OR 'comoros'/exp OR 'democratic republic congo'/exp OR 'congo'/exp OR 'cote d`ivoire'/exp OR 'equatorial guinea'/exp OR 'eritrea'/exp OR 'eswatini'/exp OR 'ethiopia'/exp OR 'gabon'/exp OR 'gambia'/exp OR 'ghana'/exp OR 'guinea'/exp OR 'guinea-bissau'/exp OR 'kenya'/exp OR 'lesotho'/exp OR 'liberia'/exp OR 'madagascar'/exp OR 'malawi'/exp OR 'mali'/exp OR 'mauritania'/exp OR 'mauritius'/exp OR 'mozambique'/exp OR 'namibia'/exp OR 'niger'/exp OR 'nigeria'/exp OR 'rwanda'/exp OR 'sao tome and principe'/exp OR 'senegal'/exp OR 'seychelles'/exp OR 'sierra leone'/exp OR 'somalia'/exp OR 'south africa'/exp OR 'south sudan'/exp OR 'sudan'/exp OR 'tanzania'/exp OR 'togo'/exp OR 'uganda'/exp OR 'zambia'/exp OR 'zimbabwe'/exp | [**115**](https://www-embase-com.proxy.library.emory.edu/) |
| **Scopus Search strategy** | Result retrieved |
| ( "implementation" OR "guideline adherence" OR "compliance" AND "drug prescription" OR "drug prescriptions" OR "antibiotic prescription" OR "antimicrobial prescription" AND "clinical practice guideline" OR "treatment protocols" OR "standard treatment guidelines" OR "antibiotic use guidelines" OR "antimicrobial use guidelines" ) OR ( Angola OR Benin OR Botswana OR Burkina AND Faso OR Burundi OR Cabo AND Verde OR Cameroon OR Central AND African AND Republic OR Chad OR Comoros OR Congo, AND Democratic AND Republic AND of OR Congo, AND Republic AND of OR Cote AND D'ivoire OR Equatorial AND Guinea OR Eritrea OR Swaziland OR Ethiopia OR Gabon OR Gambia OR Ghana OR Guinea OR Guinea-Bissau OR Kenya OR Lesotho OR Liberia OR Madagascar OR Malawi OR Mali OR Mauritania OR Mauritius OR Mozambique OR Namibia OR Niger OR Nigeria OR Rwanda OR Sao AND Tome AND Principe OR Senegal OR Seychelles OR Sierra AND Leone OR Somalia OR South AND Africa OR South AND Sudan OR Sudan OR Tanzania OR Togo OR Uganda OR Zambia OR Zimbabwe ) | [**35**](https://www-embase-com.proxy.library.emory.edu/) |

| **Web of Science Search strategy** | Result retrieved |
| --- | --- |
| TS=(Implementation* OR guideline adherence* OR guideline compliance* AND drug prescription* OR drug prescriptions* OR antibiotic prescription* OR antimicrobial prescription* AND clinical practice guideline* OR treatment protocols* OR standard treatment guidelines* OR antibiotic use guidelines* OR antimicrobial use guidelines*) AND TS=(Angola* OR Benin* OR Botswana* or Burkina Faso* or Burundi* or Cabo Verde* or Cameroon* or Central African Republic* or Chad* or Comoros* or Congo, Democratic Republic of* or Congo, Republic of* or Cote d'Ivoire* or Equatorial Guinea* or Eritrea* or Swaziland* or Ethiopia* or Gabon* or Gambia* or Ghana* or Guinea* or Guinea-Bissau* or Kenya* or Lesotho* or Liberia* or Madagascar* or Malawi* or Mali* or Mauritania* or Mauritius* or Mozambique* or Namibia* or Niger* or Nigeria* or Rwanda* or Sao Tome* and Principe* or Senegal* or Seychelles* or Sierra Leone* or Somalia* or South Africa* or South Sudan* or Sudan* or Tanzania* or Togo* or Uganda* or Zambia* or Zimbabwe*) | [**635**](https://www-embase-com.proxy.library.emory.edu/) |
| **CINAHL Search strategy** | Result retrieved |
| ( "implementation" OR "guideline adherence" OR "compliance" AND "drug prescription" OR "drug prescriptions" OR "antibiotic prescription" OR "antimicrobial prescription" AND "clinical practice guideline" OR "treatment protocols" OR "standard treatment guidelines" OR "antibiotic use guidelines" OR "antimicrobial use guidelines" ) OR ( Angola OR Benin OR Botswana OR Burkina AND faso OR Burundi OR Cabo AND Verde OR Cameroon OR Central AND African AND Republic OR Chad OR Comoros OR Congo, AND Democratic AND Republic AND of OR Congo, AND Republic AND of OR Cote AND D'ivoire OR Equatorial AND Guinea OR Eritrea OR Eswatini OR Ethiopia OR Gabon OR Gambia OR Ghana OR Guinea OR Guinea-Bissau OR Kenya OR Lesotho OR Liberia OR Madagascar OR Malawi OR Mali OR Mauritania OR Mauritius OR Mozambique OR Namibia OR Niger OR Nigeria OR Rwanda OR Sao AND Tome AND Principe OR Senegal OR Seychelles OR Sierra AND Leone OR Somalia OR South AND Africa OR South AND Sudan OR Sudan OR Tanzania OR Togo OR Uganda OR Zambia OR Zimbabwe ) | [**549**](https://www-embase-com.proxy.library.emory.edu/) |

**Appendix II: PRISMA 2020 Checklist**

| **Section and Topic** | **Item #** | **Checklist item** | **Location where item is reported** |
| --- | --- | --- | --- |
| **TITLE** | | |  |
| Title | 1 | Identify the report as a systematic review. | Page 1 |
| **ABSTRACT** | | |  |
| Abstract | 2 | See the PRISMA 2020 for Abstracts checklist. | Page 2 |
| **INTRODUCTION** | | |  |
| Rationale | 3 | Describe the rationale for the review in the context of existing knowledge. | Page 3 |
| Objectives | 4 | Provide an explicit statement of the objective(s) or question(s) the review addresses. | Page 3 & 4 |
| **METHODS** | | |  |
| Eligibility criteria | 5 | Specify the inclusion and exclusion criteria for the review and how studies were grouped for the syntheses. | Page 4 |
| Information sources | 6 | Specify all databases, registers, websites, organisations, reference lists and other sources searched or consulted to identify studies. Specify the date when each source was last searched or consulted. | Page 4 |
| Search strategy | 7 | Present the full search strategies for all databases, registers and websites, including any filters and limits used. | Page 4 |
| Selection process | 8 | Specify the methods used to decide whether a study met the inclusion criteria of the review, including how many reviewers screened each record and each report retrieved, whether they worked independently, and if applicable, details of automation tools used in the process. | Page 4&5 |
| Data collection process | 9 | Specify the methods used to collect data from reports, including how many reviewers collected data from each report, whether they worked independently, any processes for obtaining or confirming data from study investigators, and if applicable, details of automation tools used in the process. | Page 5 |
| Data items | 10a | List and define all outcomes for which data were sought. Specify whether all results that were compatible with each outcome domain in each study were sought (e.g. for all measures, time points, analyses), and if not, the methods used to decide which results to collect. | Page 5 |
|  | 10b | List and define all other variables for which data were sought (e.g. participant and intervention characteristics, funding sources). Describe any assumptions made about any missing or unclear information. | Page 6 |
| Study risk of bias assessment | 11 | Specify the methods used to assess risk of bias in the included studies, including details of the tool(s) used, how many reviewers assessed each study and whether they worked independently, and if applicable, details of automation tools used in the process. | Page 6 |
| Effect measures | 12 | Specify for each outcome the effect measure(s) (e.g. risk ratio, mean difference) used in the synthesis or presentation of results. | Page 6 |
| Synthesis methods | 13a | Describe the processes used to decide which studies were eligible for each synthesis (e.g. tabulating the study intervention characteristics and comparing against the planned groups for each synthesis (item #5)). | Page 6 |
|  | 13b | Describe any methods required to prepare the data for presentation or synthesis, such as handling of missing summary statistics, or data conversions. | Page 6 |
|  | 13c | Describe any methods used to tabulate or visually display results of individual studies and syntheses. | Page 6 |
|  | 13d | Describe any methods used to synthesize results and provide a rationale for the choice(s). If meta-analysis was performed, describe the model(s), method(s) to identify the presence and extent of statistical heterogeneity, and software package(s) used. | Page 6 |
|  | 13e | Describe any methods used to explore possible causes of heterogeneity among study results (e.g. subgroup analysis, meta-regression). | Page 6 |
|  | 13f | Describe any sensitivity analyses conducted to assess robustness of the synthesized results. | Page 6 |
| Reporting bias assessment | 14 | Describe any methods used to assess risk of bias due to missing results in a synthesis (arising from reporting biases). | Page 5 |
| Certainty assessment | 15 | Describe any methods used to assess certainty (or confidence) in the body of evidence for an outcome. | Not applicable |
| **RESULTS** | | |  |
| Study selection | 16a | Describe the results of the search and selection process, from the number of records identified in the search to the number of studies included in the review, ideally using a flow diagram. | Page 6&7 |
|  | 16b | Cite studies that might appear to meet the inclusion criteria, but which were excluded, and explain why they were excluded. | Page 6&7 |
| Study characteristics | 17 | Cite each included study and present its characteristics. | Page 6&7 |
| Risk of bias in studies | 18 | Present assessments of risk of bias for each included study. | Page 6 (Table 1) |
| Results of individual studies | 19 | For all outcomes, present, for each study: (a) summary statistics for each group (where appropriate) and (b) an effect estimate and its precision (e.g. confidence/credible interval), ideally using structured tables or plots. | Not applicable |
| Results of syntheses | 20a | For each synthesis, briefly summarise the characteristics and risk of bias among contributing studies. | Page 7&8 |
|  | 20b | Present results of all statistical syntheses conducted. If meta-analysis was done, present for each the summary estimate and its precision (e.g. confidence/credible interval) and measures of statistical heterogeneity. If comparing groups, describe the direction of the effect. | Page 7&8 |
|  | 20c | Present results of all investigations of possible causes of heterogeneity among study results. | Page 7&8 |
|  | 20d | Present results of all sensitivity analyses conducted to assess the robustness of the synthesized results. | Page 7&8 |
| Reporting biases | 21 | Present assessments of risk of bias due to missing results (arising from reporting biases) for each synthesis assessed. | Page 7&8 |
| Certainty of evidence | 22 | Present assessments of certainty (or confidence) in the body of evidence for each outcome assessed. | Not applicable |
| **DISCUSSION** | | |  |
| Discussion | 23a | Provide a general interpretation of the results in the context of other evidence. | Page 8&9 |
|  | 23b | Discuss any limitations of the evidence included in the review. | Page 8&9 |
|  | 23c | Discuss any limitations of the review processes used. | Page 9 |
|  | 23d | Discuss implications of the results for practice, policy, and future research. | Page 10 |
| **OTHER INFORMATION** | | |  |
| Registration and protocol | 24a | Provide registration information for the review, including register name and registration number, or state that the review was not registered. | Page 4 |
|  | 24b | Indicate where the review protocol can be accessed, or state that a protocol was not prepared. | Page 4 |
|  | 24c | Describe and explain any amendments to information provided at registration or in the protocol. | Not applicable |
| Support | 25 | Describe sources of financial or non-financial support for the review, and the role of the funders or sponsors in the review. | Page 4 |
| Competing interests | 26 | Declare any competing interests of review authors. | Page 10 |
| Availability of data, code and other materials | 27 | Report which of the following are publicly available and where they can be found: template data collection forms; data extracted from included studies; data used for all analyses; analytic code; any other materials used in the review. | Page 10 |
